# Effects of individual, household and community characteristics on child nutritional status in the slums of urban Bangladesh

**DOI:** 10.1186/s13690-017-0176-x

**Published:** 2017-02-20

**Authors:** Karar Zunaid Ahsan, Shams El Arifeen, Md. Abdullah Al-Mamun, Shusmita H. Khan, Nitai Chakraborty

**Affiliations:** 1 MEASURE Evaluation, Carolina Population Center, The University of North Carolina at Chapel Hill, Chapel Hill, NC USA; 2 Maternal and Child Health Division (MCHD), International Centre for Diarrhoeal Disease Research, Bangladesh (icddr,b), Dhaka, Bangladesh; 3 Health System and Population Studies Division, International Centre for Diarrhoeal Disease Research, Bangladesh (icddr,b), Dhaka, Bangladesh; 4 Department of Statistics, Biostatistics & Informatics, The University of Dhaka, Dhaka, Bangladesh

**Keywords:** Urban health, Child nutrition, Slums, Bangladesh

## Abstract

**Background:**

Bangladesh urban population is expected to overtake rural population by 2040, and a significant part of the increase will be in slums. Wide disparities between urban slums and the rest of the country can potentially push country indicators off track unless the specific health and nutrition needs of the expanding slum communities are addressed. The study aims at describing the individual, household and community determinants of undernutrition status among children living in major urban strata, viz. City Corporation slums and non-slums, in order to understand the major drivers of childhood undernutrition in urban slum settings.

**Methods:**

Data are derived from Bangladesh Urban Health Survey conducted in 2013. This survey is a large-scale, nationally representative of urban areas, household survey designed specifically to provide health and nutrition status of women and children in urban Bangladesh.

**Results:**

Data showed that 50% of under-5 children in slums are stunted and 43% are underweight, whereas for non-slums these rates are 33 and 26% respectively. In terms of severity, proportion of under-5 children living in slums severely underweight or stunted are nearly double than the children living in non-slums. Logistic analyses indicate that mother’s education, child’s age, and household’s socio-economic status significantly affects stunting and underweight levels among children living in the urban slums. Logistic models also indicate that all individual-level characteristics, except exposure to mass media and mother’s working outside home, significantly affect undernutrition levels among children living on non-slums. Among the household- and community-level characteristics, only household’s socioeconomic status remains significant for the non-slums.

**Conclusions:**

Poor nutritional status is a major concern in slum areas, particularly as this group is expected to grow rapidly in the next few years. The situation calls for specially designed and well targeted interventions that take into account that many of the mothers are poorer and less educated, which affects their ability to provide care to their children.

## Background

Globally, undernutrition was estimated to be the cause for 45% of all deaths among children under five in 2011, which translates into 3.1 million children dying every year [1]. Nonetheless this is not the end, because for those who does not die, awaits a far-reaching, long-term effects. Malnutrition – in the form of undernutrition in the first 1,000 days of any child embodies a vital squandering on future health outcomes. Unfortunately, any nutritional disorder during this period of crucial times, creates the risk of possessing irreversible damages for later life – starting from school performance, lower work capacity and productivity to have an increased likelihood of being overweight and developing associated non communicable diseases [2, 3]. Therefore, it is easily admissible that, such detrimental influences of undernutrition have potential for diminishing economic growth and hamper the goals of poverty reduction. In this regard, the global development community has recognized that the slow pace of reducing childhood undernutrition would actually create hindrance for reaching the targets related to child health and mortality for countries like Bangladesh, which are on its way to achieve many of the other global goals [4].

In the social sector, Bangladesh has made remarkable progress in many areas during the last two decades, i.e. increase in literacy and life expectancy at birth; sustaining child immunization above 90%; and achieving sharp decline of maternal mortality ratio. Gradual improvement of basic health and nutrition services contributed to substantial reduction of under-five mortality (from 94 deaths per 1,000 live births in 1999–2000 to 53 in 2011) [5], for which Bangladesh received the United Nations Millennium Development Goal (MDG) Award in 2010. However, despite these successes, nearly one-third (32%) of the population still live below the poverty line and about 25% was either unemployed or underemployed in 2010 [6, 7]. In addition to other developmental challenges, rapid urbanization and urban health are now among the major population issues facing the country – United Nations estimated that the urban population will grow by 50% during the next 14 years (2015–2029), and Bangladesh will become an urban country by 2039 when the majority of people will live in urban areas [8]. Urban populations are diverse and varied, both economically and in terms of living conditions that affect health negatively.

To a large extent urban areas are characterized by large inequalities in health-related conditions. The heterogeneity of such urban conditions is fueled by the migration process that is the primary factor of urban growth [9]. Despite making impressive progress in reducing fertility and mortality and improving health and nutrition indicators, such indicators are still lagging when it comes to urban areas – especially in terms of malnutrition. An astounding number – more than five mission – of children less than five years old have stunted growth while around 450,000 children suffer from deadly severe acute malnutrition. Based on the Bangladesh Demographic and Health Survey 2014, currently wasting or acute malnutrition affects 14% of Bangladeshi children, while one-third of children are also under weight, which is a composite of stunting and wasting [10]. The rates are equally debauched for urban Bangladesh, where half of the under-five children in slums were stunted (height-for-age below -2SD), which is around one-third for non-slums. Underweight among under-five children in slums (43%) is considerably higher in non-slums (26%) and rest urban areas (30%) [8]. This high proportion of malnutrition poses a threat to the overall progress of the country.

In 2012, the World Health Assembly (WHA) adopted a global target to reduce by 40% the number of stunted under-five children by 2025 [11]. Despite achievements, more than one-third of all children under five suffer from stunting, a condition characterized by poor linear growth and resulting from chronic malnutrition. The average annual rate of reduction (AARR) of stunting in Bangladesh is 2.7 which is much less than the required 3.3 AARR [12] to reach the global WHA target. Based on this fact, Bangladesh’s target for reducing child stunting is off track from the WHA target [13].

In this paper, we looked into the following questions. Firstly, how are the under-five children in Bangladesh doing in terms of undernutrition and which underlying and basic determinants affecting the undernutrition among children by major urban domains, viz. City Corporation slums and non-slums. Second, what are possible policy implications to tackle undernutrition in the post-MDG era in urban Bangladesh?

## Methods

### Data source

We used data from 2013 round of Bangladesh Urban Health Survey (UHS) – this is a large-scale, covering about 53,790 households, cross-sectional household survey designed specifically to provide nationally representative estimates of health and nutrition status of the urban domains like City Corporation slums and non-slums by using a three-stage sampling design [8]. For UHS, slums were defined as settlements with a minimum of 10 households with the following characteristics: very high population density and high crowding; predominantly poor housing conditions; poor water and sewerage conditions or high sharing of water sources and sewerage; and poor socioeconomic conditions [8]. UHS 2013 obtained information from ever-married women aged 13 to 49 years on about selected background characteristics; a full birth history; healthcare seeking behavior; and health, nutrition and feeding practices for children under age five years during the survey.

### Variables

Based on the terminology employed in the studies of global burden of disease and risk factors [14, 15], we opted to classify risk factors for child undernutrition as individual-, household- and community-level determinants (see Table 1).

**Table 1:** Dependent variables and selected determinants for analysis

Dependent variable	Determinants		
	Individual	Household	Community
Stunting	Mother’s age at birth of the child	Socioeconomic status	Urban domain (slum vs. non-slum)
Underweight	Mother’s educational attainment	Length of stay in the current city	Distance from health facility
	Birth order of the child	Sanitation	Availability of Community Health Worker
	Current age of the child	Garbage disposal	Location (City Corporation of Dhaka vs. the rest)
	Mother’s exposure to mass media	Household size	
	Mother working outside		
	Mother’s NGO membership		

For children alive and under age five years during the survey, weight and height were measured to calculate child nutritional status. The weight and height measurements were converted into three summary indices of nutritional status: weight-for-age, height-for-age and weight-for-height. These nutritional status measurements were evaluated against the World Health Organization Child Growth Standards [16]. Specifically, these nutritional indicators are expressed in standard deviations (Z-scores) from the mean of the standard population – children with measurements less than −2 Z-scores were considered to have undernutrition (i.e. stunting, wasting or underweight).

We categorize all dependent and independent variables to perform a bivariate analysis. Wealth quintile is considered as socio-economic status which is categorized into poor and non-poor. Since Dhaka is the city of Bangladesh and mostly covered by urban area, hence we are interested to know the variation of child nutrition between Dhaka division and other than Dhaka division by Region variable. Exposure to media variable has two categories-daily and less than daily. If child’s mother is not exposed to media (paper, radio or TV) daily, then she is categorized to “less than daily”. We focus on types of sanitation rather than its share. Improved and non-improved are two types of sanitation system.

### Statistical analysis

**Fig. 1:**
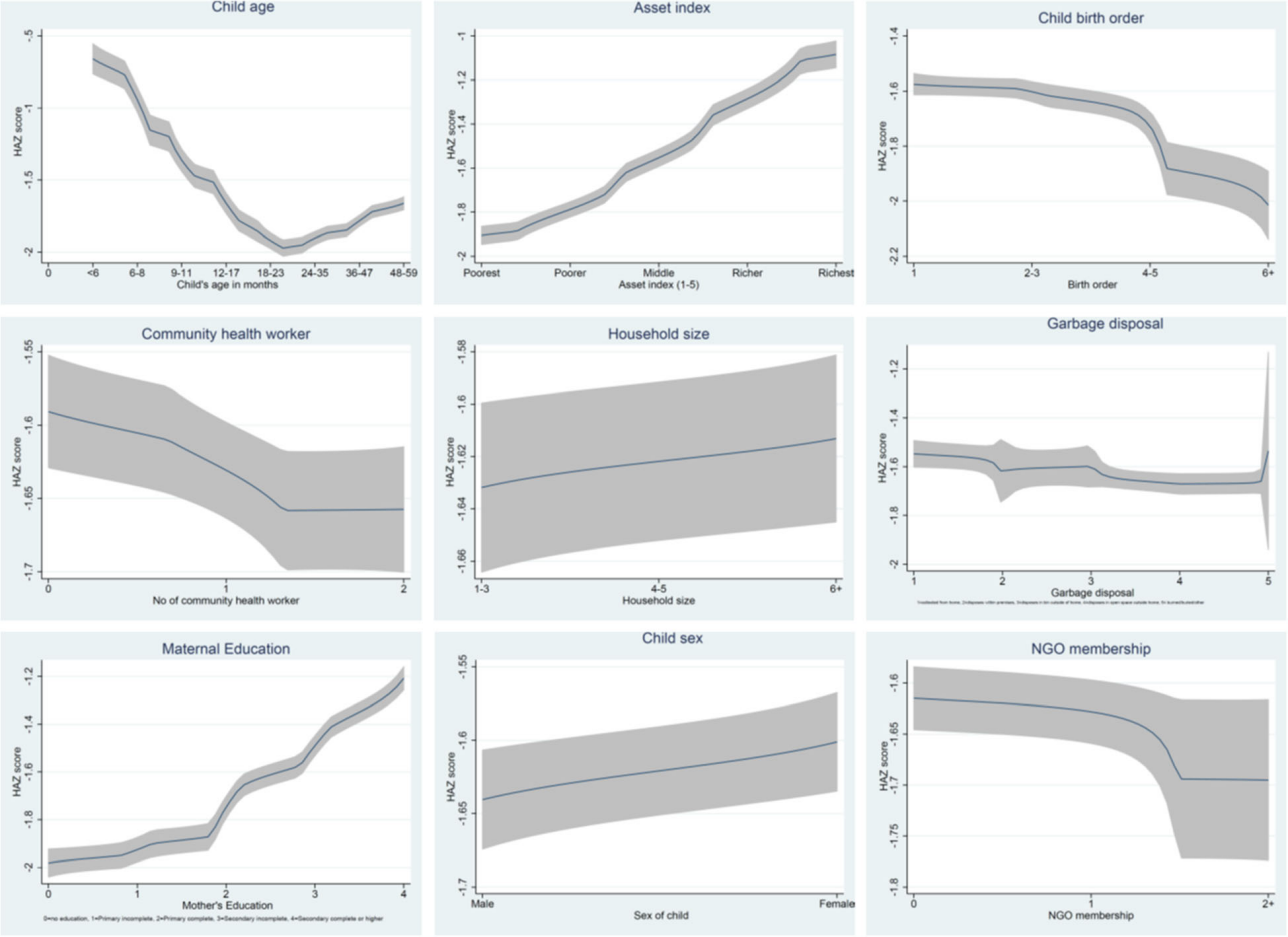
Non-parametric estimates of the relationship between HAZ score and selected determinants. Non-parametric graphical distribution of selected determinants by nutritional index (standardized height-for-age z [HAZ] score). Majority of the determinants have approximately non-linear relationships with the nutritional index

**Fig. 2:**
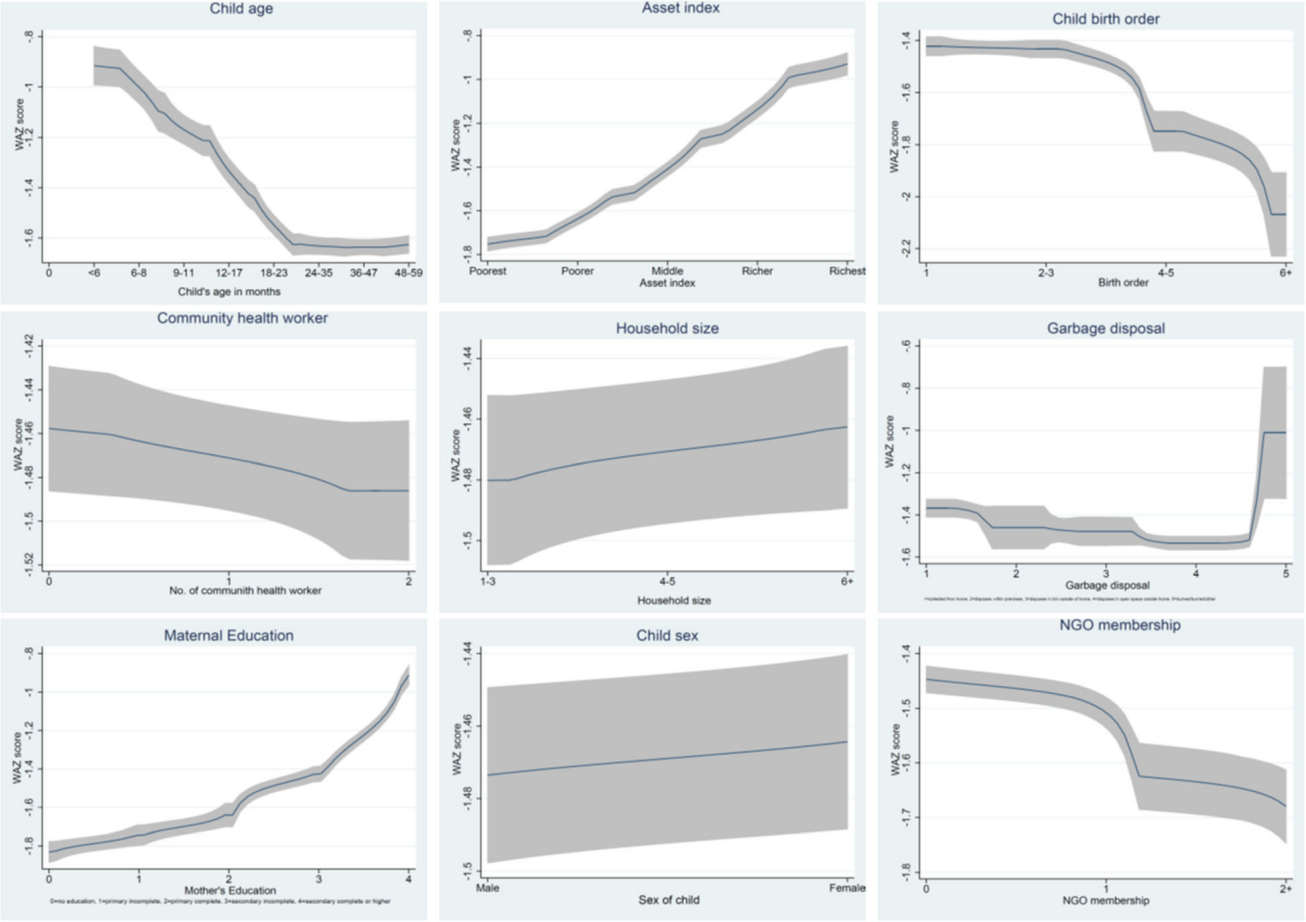
Non-parametric estimates of the relationship between WAZ score and selected determinants. Non-parametric graphical distribution of selected determinants by nutritional index (standardized weight-for-age z [WAZ] score). Majority of the determinants have approximately non-linear relationships with the nutritional index

Using data from 2013 UHS, we carried out bivariate analyses of selected health outcomes and healthcare utilization indicators in urban slums and non-slums, and illustrated how the selected predictors of child undernutrition vary over the urban domains. In order to select the appropriate statistical model to explore relationships between children’s undernutrition status and a range of individual-, household- and community-level determinants, we undertook a non-parametric graphical analyses of selected variables to examine the nature of relationship between nutritional indices (i.e. height-for-age z score [HAZ] for stunting and weight-for-age z score [WAZ] for underweight) and selected determinants. We performed Kernel-weighted local polynomial non-parametric regression which is based on smoothing [17]. Figures 1 and 2 show that majority of the determinants have approximately non-linear relationships with the nutritional indices.

Based on the relationship between summary nutritional indices and selected determinants, we conducted a logistic regression analysis on the UHS data file to explore relationships between the nutritional status and a range of individual-, household- and community-level determinants. All statistical analyses were carried out using the Stata statistical software, version 13.1 [18].

## Results

**Fig. 3:**
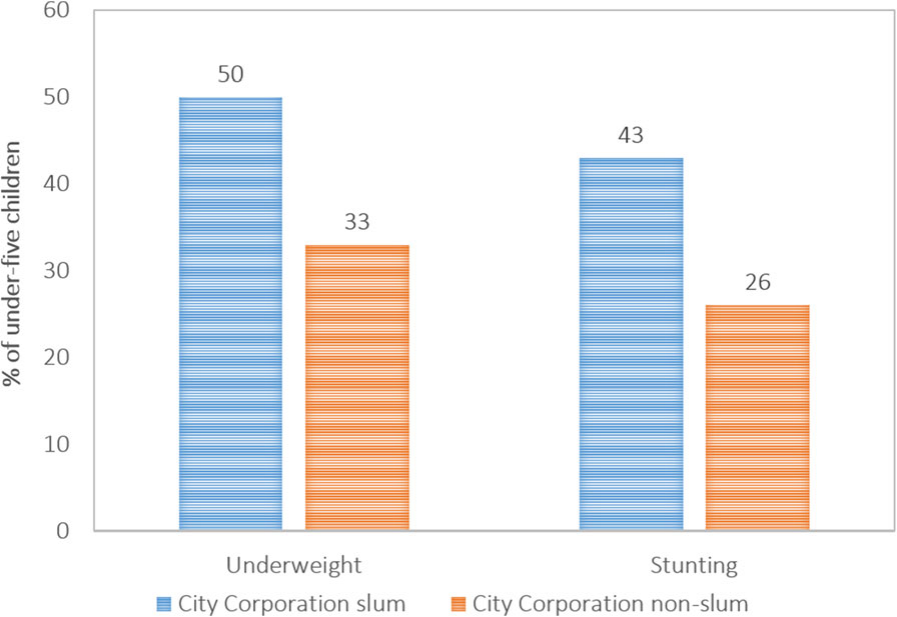
Undernutrition status of under-five children of urban Bangladesh, 2013. Distribution (%) of stunting and underweight among under-five children in Bangladesh by major urban domains, based on data from 2013 Urban Health Survey. Half of all under-5 children in City Corporation slums were stunted (50%), whereas one-in-three under-5 children in City Corporation non-slums were stunted (33%). Also, 43% of under-5 children in City Corporation slums were underweight, against 26% in City Corporation non-slum areas

Data from 2013 Urban Health Survey showed that half of all under-5 children in slums were stunted (50%), whereas one-in-three under-5 children in non-slums were stunted (33%). Also, 43% of under-5 children in City Corporation slums were underweight, against 26% in City Corporation non-slum areas. In terms of severity of underweight, proportion of under-5 children severely underweight in slums is more than double than that of the non-slums (15 and 7%, respectively). Nearly one in every four children in slums was severely stunted, which was one in seven children for non-slums (see Fig. 3). Table 2 shows the percentage of under-five children who are stunted and underweight by the selected determinants in slums and non-slums area in urban Bangladesh.

**Table 2:** Distribution (%) of undernutrition status among under-5 children by selected background characteristics

Background characteristics	Stunting		Underweight	
	Slums	Non-slums	Slums	Non-slums
Mother’s age at birth				
< 20 years	53.1	35.6	46.0	33.6
20–34 years	48.2	33.1	41.1	25.2
35+ years	51.1	32.5	42.7	21.6
Birth order				
1	50.9	30.0	43.7	26.5
2–3	47.6	35.4	40.9	25.1
4+	52.7	41.7	44.3	36.0
Region				
Other Division	47.3	31.7	42.9	26.9
Dhaka	51.1	34.5	42.2	26.1
Mother’s education				
No education	53.1	47.7	46.2	40.5
Primary	53.2	41.9	46.0	33.3
Secondary+	42.8	29.9	35.6	23.4
Child’s age in months				
< 9	26.4	22.5	26.0	16.9
9–23	53.6	39.3	38.8	25.4
24–59	52.7	33.4	47.7	29.0
Wealth Quintile				
Non-poor	42.3	30.4	34.5	23.4
Poor	52.1	43.6	45.2	36.6
Distance from health facility				
< 1 km	49.9	33.1	42.1	26.1
1–2 km	49.1	34.7	44.7	27.6
> 2 km	49.3	33.4	34.0	26.3
Number of available community health worker				
None	49.9	32.7	41.8	26.2
One	48.3	31.7	41.6	24.5
Two or more	50.2	36.5	43.8	28.4
Land ownership				
Personal	49.3	33.5	42.3	26.5
Government	53.3	31.5	44.5	21.6
Duration of residence				
< 2 years	51.1	30.7	46.5	31.1
2–5 years	47.7	35.2	41.7	27.7
> 5 years	49.8	33.4	42.2	25.8
Exposure to media (paper, radio or TV)				
Less than daily	54.1	42.8	46.7	37.5
Daily	47.8	32.4	40.7	25.2
Household size				
1–3	48.9	34.7	41.0	29.0
4–5	50.1	33.5	43.3	24.8
6+	49.4	32.6	42.2	27.4
Sanitation facility				
Not-improved	54.3	33.4	46.0	25.5
Improved	48.7	33.5	41.8	26.7
Garbage disposal				
In open space	50.8	35.9	43.6	31.3
Disposed	48.2	32.4	41.1	24.2
NGO’s membership				
Not member	50.2	32.5	42.4	25.6
Member	46.7	46.3	43.2	36.8
Mother’s working status				
Unemployed/works from home	48.5	32.7	40.8	26.4
Works outside home	54.7	40.7	49.9	26.3
Total observations	5,027	2,559	5,231	2,657

Logistic analysis indicates (Table 3) that individual characteristics like maternal education, age of the child, and media exposure have statistically significant impact on the likelihood of stunting among under-five children living in slums of urban Bangladesh. Mothers’ educational status strongly affects the likelihood of undernutrition among children – children of mothers having secondary education incomplete or higher education are nearly 33% less likely to be stunted or underweight, compared with mothers with no education. Children of the mothers with daily exposure to mass media also are nearly 20% less likely to be stunted or underweight compared with mothers with irregular exposure. Also as the child gets older, likelihood of being stunted increases by more than three-fold (and nearly twice for underweight) compared with children aged less than 9 months. Apart from these characteristics, mother’s working outside home significantly increases the likelihood of underweight among children living in slums.

**Table 3:** Regression estimates on undernutrition status among children living in urban slums, 2013

Background Characteristics	Stunting				Underweight			
	Individual- level	Household- level	Cluster- level	Model A	Individual- level	Household- level	Cluster- level	Model B
Mother’s age at birth (reference: <20 years)								
20-34 years	0.85				0.84			
35+ years	0.84				0.83			
Mother’s education (reference: no education)								
Primary	1.03			1.05	1.03			1.07
Secondary +	0.67^***^			0.74^**^	0.68^***^			0.76^**^
Birth order (reference: 1)								
2-3	0.90				0.90			
4+	0.99				0.93			
Child’s age (reference: <9 months)								
9-23 months	3.30^***^			3.31^***^	1.79^***^			1.82^***^
24-59 months	3.13^***^			3.15^***^	2.50^***^			2.57^***^
Exposure to media (paper, radio or TV) (reference: less than daily)								
Daily	0.81^*^			0.86	0.83^*^			0.88
Mother’s working status (reference: unemployed/works from home)								
Outside home	1.07				1.21^*^			1.16
NGO’s membership (reference: not member)								
Member	0.85				1.03			
Wealth status (reference: non-poor)								
Poor		1.49^***^		1.31^**^		1.57^***^		1.36^**^
Duration of residence (reference: <2 years)								
2-5 years		1.09				0.98		
>5 years		1.03				0.96		
Sanitation facility (reference: not improved)								
Improved		0.80^*^		0.81		0.84		0.84
Garbage disposal (reference: in open space)								
Disposed		0.96				0.98		
Household size (reference: 1-3)								
4-5		1.08				1.15		
6+		1.09				1.14		
Distance from health facility (reference: <1 km)								
1-2 km			0.96				1.11	1.12
>2 km			0.94				0.72	0.65^*^
Number of available community health worker (reference: none)								
One			0.88				0.98	
Two or more			0.95				1.07	
Region (reference: other division)								
Dhaka			1.19^*^	1.13			0.96	

Exponentiated coefficients; ^*^ *p* < 0.05, ^**^ *p* < 0.01, ^***^ *p* < 0.001

In terms of household characteristics, socio-economic status and sanitation facility have statistically significant impact on the nutritional status of children living in slums of urban Bangladesh. Children from poor households are 49% more likely to be stunted and 57% more likely to be underweight compared with the children from non-poor households. For slums households with improved sanitation facilities, children are 20% less likely to be stunted compared with children living in households without improved sanitation facility. None of the community characteristics (i.e. cluster-level variables) considered in this analysis, except location, significantly affects undernutrition status among children living in the urban areas. Children living in slums within Dhaka City Corporation are 19% more likely to be stunted compared with the children living in slums in the other City Corporations in the country. No significant pattern was observed for household’s distance from health facility or availability of community health workers on the child’s undernutrition status in the analysis.

The models A and B in Table 3 include all the variables that achieved a significance level of 0.10 or better in the separate logistic models for individual-, household-, and cluster-level characteristics for stunting and underweight respectively among children living in the urban slums. The models thus includes several individual-level variables, two household-level variables and a cluster-level variable. The final model for stunting (Model A) indicates that mother’s education, child’s age, and household’s socio-economic status significantly affects stunting level among children living in the urban slums. Children of mothers having secondary education or higher education are 24% less likely to be stunted, compared with mothers with no education. Children’s age and household’s socioeconomic status continue to strongly affecting the likelihood of stunting among children. However, regular exposure to mass media and improved sanitation facility at the household level remain protective against stunting but loses significance. The effect of region also loses its significance in the final model for stunting. Also in the final model for underweight (Model B), mother’s education, child’s age, and household’s socio-economic status significantly affects underweight level among children living in the urban slums. Interestingly, distance from the nearest health facility significantly lowers the likelihood of being underweight among children living in slums.

Logistic models also indicate that the undernutrition status among children living in the City Corporation non-slums are predominantly affected by individual-level characteristics (Table 4). Except mothers’ working outside home, all other individual-level variables included in the regression models found to be significant (only regular exposure to mass media remains significantly protective against underweight but not stunting). Unlike the children living in urban slums, mother’s age at birth and birth order significantly affects the undernutrition status of children living in the non-slums. Outside the individual-level characteristics, only low socioeconomic status significantly increases the likelihood of being undernourished (nearly 80% more than the children from non-poor households). Also, proper garbage disposal from households reduced the likelihood of being underweight by 20%.

**Table 4:** Regression estimates on undernutrition status among children living in urban non-slums, 2013

Background Characteristics	Stunting				Underweight			
	Individual- level	Household-level	Cluster- level	Model C	Individual- level	Household-level	Cluster- level	Model D
Mother’s age at birth (reference: <20 years)								
20-34 years	0.76^*^			0.78	0.66^**^			0.68^**^
35+ years	0.54^*^			0.58^*^	0.40^***^			0.43^**^
Mother’s education (reference: no education)								
Primary	0.85			0.87	0.78			0.80
Secondary +	0.56^**^			0.62^**^	0.53^***^			0.60^**^
Birth order (reference: 1)								
2-3	1.36^**^			1.35^**^	1.04			1.05
4+	1.52^*^			1.49	1.50			1.47
Child’s age (reference: <9 months)								
9-23 months	2.30^***^			2.30^***^	1.68^**^			1.67^**^
24-59 months	1.73^***^			1.73^***^	2.02^***^			2.01^***^
Exposure to media (paper, radio or TV) (reference: less than daily)								
Daily	0.85				0.71^*^			0.84
Mother’s working status (reference: unemployed/works from home)								
Outside home	1.34			1.33	0.88			
NGO’s membership (reference: not member)								
Member	1.57^**^			1.51^*^	1.49^*^			1.38
Wealth status (reference: non-poor)								
Poor		1.80^***^		1.35^**^		1.79^***^		1.32^*^
Duration of residence (reference: <2 years)								
2-5 years		0.96				0.98		
>5 years		1.15				0.88		
Sanitation facility (reference: not improved)								
Improved		0.96				0.96		
Garbage disposal (reference: in open space)								
Disposed		1.00				0.80^*^		0.84
Household size (reference: 1-3)								
4-5		1.01				0.87		
6+		1.01				1.06		
Distance from health facility (reference: <1 km)								
1-2 km			1.08				1.08	
>2 km			1.06				1.02	
Number of available community health worker (reference: none)								
One			0.94				0.93	
Two or more			1.18				1.13	
Region (reference: other division)								
Dhaka			1.14				0.96	

Exponentiated coefficients; ^*^ *p* < 0.05, ^**^ *p* < 0.01, ^***^ *p* < 0.001

When all the variables that achieved a significance level of 0.10 or better in the separate logistic models for individual-, household-, and cluster-level characteristics for undernutrition were fitted in the regression models (model C and D in Table 4) for the children living in the non-slums, all individual-level characteristics except exposure to mass media and mother’s working outside home remained significant. Among the household- and community-level characteristics, only household’s socioeconomic status remains significant in the final models.

## Discussions

Understanding the nutritional status of children living in urban areas, with a focus on slums, is imperative for a comprehensive approach to tackle malnutrition. Evidence suggests that under-five children living in slums are even more malnourished than their counterparts living in rural areas. Meaning, migrating to urban area – mostly for better livelihood – does not necessarily improve the undernutrition situation among the children of the poorer population groups. For Bangladesh, urban population is expected to overtake rural population by 2040, and a significant part of the increase will be in slums. Wide disparities between urban slums and the rest of the country can potentially push country indicators off track unless the specific health and nutrition needs of the expanding slum communities are addressed.

The results of this study show high levels of undernutrition among children living in urban slums, and this finding coincides with other studies from this region [19–21]. Looking into the cause of high this proportion of undernutrition, studies claimed that immediate causes of undernutrition are malnutrition in utero, inadequate infant and young child feeding practices and early life infections; followed by the intermediate and underlying factors including (but are not limited to) child care practices, food security, household wealth, maternal education, health services, hygiene practices and sanitation/hygiene conditions [22–24]. Also, the consequence of other household attributes, such as female literacy [25], empowerment [26], and maternal health knowledge [27] are being acknowledged.

The analyses carried out in this study validated the role of the majority of these immediate and underlying determinants of childhood malnutrition in the urban areas of Bangladesh. Whilst the impact of mother’s age at birth on stunting was not found significant for slums, unlike other studies in the region [28], children of younger mothers are more likely to be stunted in the non-slums. This analysis found that mothers’ educational level significantly affects malnutrition among children, which is consistent with studies showing that maternal education is a strong predictor of child stunting after adjusting other factors at individual, household and community levels [29–32]. Though higher educational attainment should increase the likelihood of mothers’ working outside home, the study sample indicates that mothers’ engagement in working outside home was low (overall 13% mothers worked outside home, 19% in slums and 9% in non-slums) and poverty, rather than educational attainment, primarily drove mothers to work outside. Logistic regression model at individual-level found that mothers’ working outside home significantly increased childhood underweight, but it lost significance in the final model after adding household’s wealth status.

Child’s birth order and size of the family has a strong relationship with child’s nutrition status, especially in case of children from slums. This matches with other studies showing that likelihood of children being malnourished has strong association with parity [33, 34]. Younger children being more likely to be malnourished (stunted) was also found in our analysis, which coincides with other papers looking into urban children’s nutrition status [35–37]. Other household and societal factors like mother’s exposure to mass media, NGO membership/access to social entities also plays an important role for determining the nutritional status of the children [38, 39].

Our study indicates that, children from poor families are more likely to be more malnourished, which coincides with other evidences. In developing countries, as predicted children from poorer section of the community are more prone to be malnourished [40]. For people living in slums, poverty is perceived with income and consumption patterns. In majority cases, poor are involved in stumpy earning jobs and often has inadequate income to support their basic needs. The truncated level of earning of the urban poor subsequently results in spending majority of the earnings on food, mainly staples like rice, cereals, lentils, potatoes and vegetables, and usually evade costly items like meat and poultry, milk and fruits. There are school of thoughts, that indicates that, chronic malnutrition and undernutrition among children is often the result of deprivation of such necessary food items over a long period of time [41, 42]. For Bangladesh, Dhaka consists the largest proportion of poor urban inhabitants [43] and almost half of the poor households of Dhaka’s slums are hardcore poor [44, 45]. This may explain the higher proportion of children living in Dhaka slums to be more undernourished.

Our analysis came up with some interesting results, particularly on the significant protective effects of media exposure and sanitation facilities in slum settings, and significant protective effects of media exposure and garbage disposal in non-slums settings – children living in the slums with improved sanitation facilities are less likely to be stunted or underweight, and children living in the non-slums with proposer garbage disposal at household levels are less likely to be underweight. This supports the recent evidence that suggests that environmental contamination causes growth faltering mediated through environmental enteropathy [46, 47]. This condition develops when young children are repeatedly exposed to pathogenic bacteria that colonize the normally sterile small intestinal mucosa. Environmental enteropathy sets up a chronic inflammation in the small intestinal mucosa, damaging the mucosal villi that absorb nutrients and preventing proper absorption of nutrients leading to malnutrition.

### Recommended interventions for improving nutritional status of children in urban slums

In order to effectively address the high undernutrition among under-five children in urban Bangladesh, a multipronged approach would be required involving both the social development and nutrition-based interventions. First, this study shows that maternal factors significantly affect a child’s nutritional status, thus improvement in the social status of women by increasing age at birth and maternal educational status will have a positive impact on nutrition status of the children [28, 48]. Efforts directed towards improvement of women empowerment and restricting family size will also play a crucial role in addressing undernutrition among children living in slums. Second, results of our analysis suggests that it is the environment in a slum setting littered with pathogenic bacteria that predisposes a child to environmental enteropathy. To prevent this condition, we need adequate planning and sustained investment for improving personal hygiene, functional latrines, and proper garbage disposal in the City Corporation slum areas. Third, specific nutrition-specific as well as nutrition-sensitive interventions needs to be implemented targeting slum population, which have demonstrated evidence for reducing childhood undernutrition. Some of the priority interventions for urban slums, based on this study’s findings, are highlighted below:

1. **Nutrition education:** Need-based nutrition education and behavioral change communication for the family has been effective in favorably changing feeding practices in urban slums [49]. Studies indicate that nutrition education activities promote early and exclusive breastfeeding as well as complementary feeding among infants, particularly when the families are supported by enabling social networks [15, 50]. Study on urban slums of Bangladesh also suggests that nutrition education program has stronger impact in improving the nutritional status of children compared with targeted food supplementation programs [51].
2. **Infection control in urban communities:** In slum settings, children are especially susceptible to undernutrition due to environmental enteropathy. Improving drainage and sanitation facilities, and proper garbage disposal are the major nutrition-sensitive interventions with strong level of evidence. Also, deworming found to be an intervention that improves micronutrient status among children and adolescents [50, 52].
3. **Capacity building of service providers:** Trained service providers and community workers do not only enhance access to healthcare for the entire community but also can identify undernutrition and deliver nutrition education to mothers and children in the communities like urban slums, where public healthcare is absent [48, 53]. The community health workers need to be equipped with knowledge and skills to implement the nutrition components of the health program efficiently.
4. **Multi-sectoral approach:** Poverty stands out to have a major impact on undernutrition level among children lining in urban slums of Bangladesh. In order to address this, we need to link integrated health and nutrition services to social transfer mechanisms for the ultra-poor households. Bangladesh itself provides multiple examples of effective social transfer programs that have significantly improve nutritional outcomes – like food-for-work projects of the Government [54]; asset transfer and cash stipend by an non-government organization [55]; cash-for-work project of the Chars Livelihoods Program [56], etc. Based on these experiences, Bangladesh needs to design and implement appropriate social transfer programs targeted for ultra-poor households for urban slums.

## Conclusions

In the context of Bangladesh, this is the first attempt to explore the determinants of undernutrition in major urban domains using representative sample. In summary, almost half of all under-5 children in slums were undernourished, and in terms of severity, the proportion of children severely undernourished in slums is nearly double than that of the non-slums. Poor nutritional status is a major concern in slum areas, particularly as this group is expected to grow rapidly in the next few years. The situation calls for specially designed and well targeted interventions that take into account that many of the mothers are poor and less educated, which affects their ability to provide care to their children. Apart from effective nutrition-specific interventions, Bangladesh needs to adopt multi-sectoral approach to tackle undernutrition in the post-MDG era in urban Bangladesh.

## Abbreviations

AARR: Average annual rate of reduction
MDG: Millennium development goal
SD: Standard deviation
UHS: Urban health survey
WHA: World Health Assembly
